# Intrathecal lactate to predict spinal cord ischemia in major abdominal surgery

**DOI:** 10.1186/cc13639

**Published:** 2014-03-17

**Authors:** G Landoni, M Pieri, V Testa, S Silvetti, M Zambon, G Borghi, M Azzolini, AL Di Prima, L Nobile, R Lembo, A Zangrillo

**Affiliations:** 1 Vita-Salute San Raffaele University, Milan, Italy

## Introduction

The aim was to evaluate the role of intrathecal lactate as an early predictor of spinal cord injury during thoracoabdominal aortic aneurysmectomy. Forty-four consecutive patients were scheduled to undergo thoracoabdominal aortic aneurysmectomy. Two patients had a type B dissecting aneurysm; all other 42 patients suffered from degenerative aneurysm.

## Methods

During surgery, samples of cerebrospinal fluid and arterial blood were simultaneously withdrawn to evaluate lactate concentration. Samples were collected at five fixed times during and after surgery: T1 (beginning of the intervention), T2 (15 minutes after aortic cross-clamping), T3 (just before unclamping), T4 (end of surgery), and T5 (4 hours after the end of surgery).

## Results

Mean lactate levels in cerebrospinal fluid rose consistently from the beginning of the intervention steadily until after surgery (T1 = 1.83 mmol/l, T2 = 2.10 mmol/l, T3 = 2.72 mmol/l, T4 = 3.70 mmol/l, T5 = 4.31 mmol/l). Seven patients developed spinal cord injury; two of them had delayed injury occurring 24 hours after the end of surgery; the remaining five had early onset. In this group of five patients, preoperative cerebrospinal fluid lactate levels were significantly *(P *= 0.04) higher than those of the other 40 patients preoperatively (2.12 ± 0.35 vs. 1.79 ± 0.29 mmol/l).

## Conclusion

The preoperative cerebrospinal lactate concentration is elevated in patients who will develop early-onset spinal cord injury after thoracoabdominal aortic aneurysmectomy. This may allow a better stratification of these patients, suggesting a more aggressive strategy of spinal cord function preservation and possibly guaranteeing them a better outcome.

